# Chemotherapy-Induced Dysgeusia and Its Perverse Consequences: A Case Report

**DOI:** 10.7759/cureus.27908

**Published:** 2022-08-11

**Authors:** Francisco Pombo, Carolina Seabra, Ana João Sá, Inês Ferreira

**Affiliations:** 1 Internal Medicine, Centro Hospitalar do Tâmega e Sousa, Penafiel, PRT

**Keywords:** quality of life, diabetes mellitus, polydipsia, dysgeusia, chemotherapy

## Abstract

Dysgeusia is one of the most common side effects of chemotherapy. Still, there is little information given to patients and limited knowledge about its diagnosis and management. We report the case of a patient under a standard regimen of adjuvant chemotherapy treatment (cisplatin and vinorelbine) who developed a life-threatening case of diabetes mellitus decompensation (hyperosmolar hyperglycemic state) resulting from extreme dietary intake due to severe dysgeusia and polydipsia. Dysgeusia is associated with a wide range of chemotherapy drugs. It is a frequent side effect but often overlooked. Self-care strategies and pharmacological agents can be implemented to help ensure better compliance to cancer treatment and improve quality of life.

## Introduction

Dysgeusia is one of the most common side effects of chemotherapy. It leads to a lower quality of life and a complex interaction between the biological, psychological, social, and cultural relationship that unites people and food.

It is an important aspect of the quality of life for patients with cancer, being one of the most common side effects in patients going through chemotherapy. However, there is little information given to them prior to initiating treatment and limited knowledge about its management, which includes both self-care strategies and pharmacological agents [1,2].

Its prevalence has been remarkably difficult to estimate. In a systematic review, dysgeusia was reported in a broad range from 14% to 100% of patients receiving chemotherapy and radiotherapy. The severity depends upon the type of cancer and the type of treatment, being higher in cancer of the head and neck [2,3].

We describe a case of a 67-year-old male of European origin with arterial hypertension, type 2 diabetes mellitus, heart failure with preserved ejection fraction due to ischemic heart disease, permanent atrial fibrillation, and lung adenocarcinoma on the superior left lobe. The patient was diagnosed with stage 2 lung adenocarcinoma four months prior and underwent complete resection via superior left lung lobectomy and was under a standard regimen of adjuvant chemotherapy treatment with cisplatin and vinorelbine (two cycles completed).

## Case presentation

**Figure 1:**
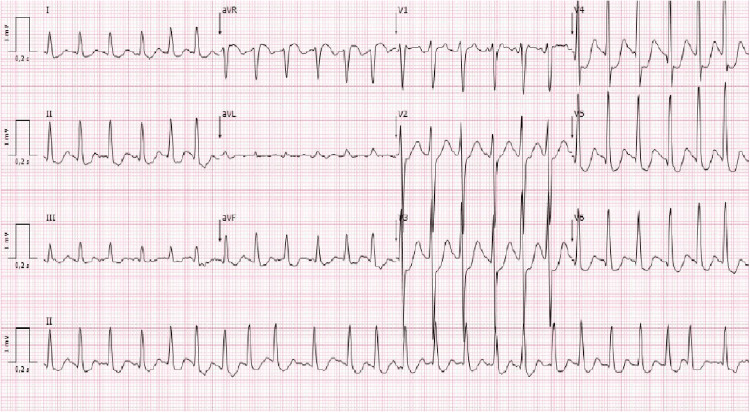
Electrocardiogram in the emergency department showing atrial fibrillation with a rapid ventricular response.

The patient was brought into the emergency department (ED) with altered mental status. He was found unresponsive in his home two days after completing the second chemotherapy cycle. On evaluation in the ED, he was unresponsive to sound and painful stimuli, had no verbal response, and had responsive and symmetrical pupils. His blood pressure was 101/55 mmHg, pulse was 126 bpm, and respiratory frequency was 26 bpm, with a normal temperature (36.6°C). A blood gas test sample was drawn, which showed metabolic and respiratory acidosis and hyperlactacidemia (5.5 mmol/L). An electrocardiogram exhibited atrial fibrillation with a rapid ventricular response (Figure 1). Laboratory results (Table 1) revealed marked hyperglycemia (2,255 mg/dL), acute renal injury (urea of 59 mg/dL and creatinine of 2.02 mg/dL), hypernatremia (184 mmol/L), and hypokalemia (2.4 mmol/L). A head computed tomography showed no acute abnormalities. Urinalysis revealed no ketone body presence, and a urological ultrasound showed no evidence of urinary obstruction. Serum osmolality was 343 mOsm/kg. As such, a diagnosis of hyperosmolar hyperglycemic state was made, and the patient was admitted to the intensive care unit (ICU).

**Table 1:** Laboratory results at admission to the emergency department. paO2: arterial partial pressure of oxygen; paCO2: arterial partial pressure of carbon dioxide

	At admission	Reference range
pH	7.16	7.35-7.45
paO2	72 mmHg	80-100 mmHg
paCO2	53 mmHg	35-45 mmHg
Bicarbonate	18.9 mmol/L	22-28 mmol/L
Lactate	5.5 mmol/L	0.6-2 mmol/L
Glycemia	2,255 mg/dL	76-110 mg/dL
Urea	56 mg/dL	10-50 mg/dL
Creatinine	1.89 mg/dL	0.81-1.44 mg/dL
Sodium	184 mmol/L	135-145 mmol/L
Potassium	2.4 mmol/L	3.5-5 mmol/L
Serum osmolality	343 mOsm/kg	285-295 mOsm/kg

**Figure 2:**
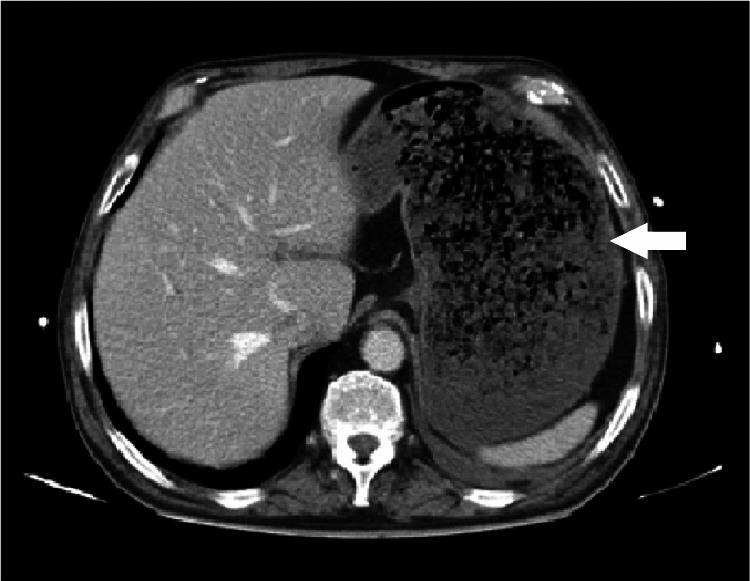
Gastric distension from liquid intake (white arrow).

While in the ICU, the patient recovered rapidly with intravenous insulin, aggressive fluid therapy, and ion supplementation with glycemic control and normalization of kidney function and diuresis. In search of an etiology for such a severe state, a full-body computed tomography was performed, which revealed a marked liquid and gas distension of the stomach and colon indicating, as the first diagnostic hypothesis, abundant liquid intake and, as the second, a gastric bezoar (Figure 2). Upper gastrointestinal endoscopy revealed no pathological findings, excluding the second hypothesis.

**Figure 3:**
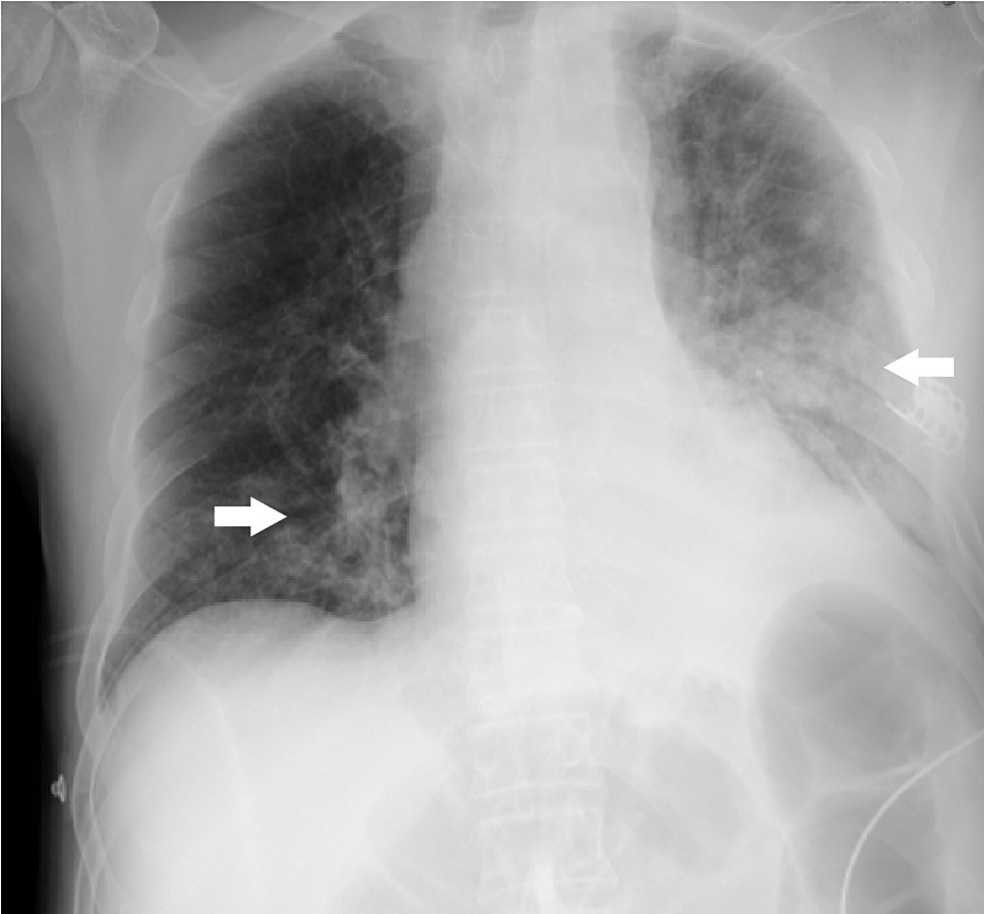
Chest X-ray showing pneumonia (white arrows).

As a major complication, the patient was diagnosed with aspiration pneumonia with severe respiratory failure with complete resolution after one cycle of empiric antibiotic therapy with amoxicillin/clavulanic acid (Figure 3).

The patient was transferred to the internal medicine department where a complete medical history with the patient fully aware was taken. The patient revealed that after the last chemotherapy session (two days before ED admission), he developed severe dysgeusia (with an intense metallic taste and burning sensation of the tongue) and polydipsia and felt an urge to drink a lot of cold sugary liquids. So, he drank a large number of soft drinks (about 11 L), to which he added a large amount of sugar. He described that the symptoms progressively worsened, which increased his impulse for liquid intake until he was found unresponsive by a family member and brought to the hospital.

The patient had a history of type 2 diabetes mellitus, diagnosed 10 years prior. He had good metabolic control (HbA1c of 5.2%, four months prior to hospitalization) treated with oral antidiabetics (metformin and dapagliflozin). In the current hospitalization, his HbA1c was 10.3%, indicating deteriorating control at least since his cancer diagnosis. Insulin therapy with a long-acting insulin analog (glargine insulin) was initiated with good tolerance. He was evaluated by a nutritionist and given personalized dietary guidelines for glycemic control and recommendations to ease chemotherapy symptoms such as dysgeusia (avoiding hot foods, preferring cold foods and drinks, avoiding sugary drinks, preferring drinks with a lemon or other fruit juices, and avoiding metal silverware and preferring plastic).

After discharge from the hospital, the patient revoked his informed consent to continue chemotherapy.

## Discussion

In this case, we describe a life-threatening decompensation of diabetes mellitus (hyperosmolar hyperglycemic state) resulting from extreme dietary intake due to severe dysgeusia and polydipsia owing to chemotherapy.

Hyperosmolar hyperglycemic state is an acute decompensation with a varied clinical presentation ranging from mild to severe. It is characterized by severe hyperglycemia, hyperosmolarity, and severe dehydration and is associated with a high mortality rate. It usually occurs in the context of infection or poor diabetic treatment compliance. However, overconsumption of a carbohydrate-rich diet can also trigger this condition. An important feature of this patient is the fact that its glycosylated hemoglobin doubled in a matter of four months. This translates to extremely poor glycemic control. However, this decompensation began at the start of chemotherapy. As a matter of fact, cisplatin has been associated with hyperglycemia and even rare cases of hyperosmolar hyperglycemic state [4]. Due to this fact, we believe that cisplatin possibly played a central role in this case, predisposing the patient to a state of permanent hyperglycemic decompensation that finally led to a severe condition triggered by excessive dietary intake.

Dysgeusia is associated with a wide range of chemotherapy drugs. The exact etiological mechanisms are still unknown; however, there are a few possible causes, such as damage to taste and olfactory cells (these are cells with higher turnover rates, usual targets for chemotherapy), neurotoxicity, and xerostomia. Radiotherapy is also associated with the disruption of the structure of taste buds and oral epithelium. Stomatitis and mucositis also play an important role [1]. In this case, the patient was under adjuvant chemotherapy treatment with cisplatin and vinorelbine. This combination has been associated with peripheral sensory neuropathy, oral mucositis, and dysgeusia (the fourth and fifth most common side effects after nausea, fatigue, anorexia, constipation, and alopecia) [5].

Treatment strategies can be divided between self-care strategies and pharmacological agents. In the first step, there are several different recommendations to improve flavors, such as consuming cold foods (such as frozen fruits); avoiding metallic silverware, red meats, coffee, or chocolate; adding more seasonings or fats to meal preparation; and drinking more water. As for pharmacological agents, zinc supplementation has been shown to improve gustatory sensation, with limited benefit. The mechanism is still not entirely understood, but zinc is a cofactor of alkaline phosphatase, which is an abundant enzyme in the taste buds [1,2,6].

## Conclusions

In summary, it is of pivotal importance to offer comprehensive information to the patient and family before the start of treatment and continuous education, counseling, and support to improve quality of life and ensure better compliance with cancer treatment. In this case, the patient revoked his informed consent for chemotherapy, proving the importance this particular episode had on his well-being and belief in the treatment, worsening his overall prognosis.
